# Data on nitrate and nitrate of Taham dam in Zanjan (Iran)

**DOI:** 10.1016/j.dib.2018.01.055

**Published:** 2018-02-02

**Authors:** Mohammadreza Massoudinejad, Mansour Ghaderpoori, Ali Jafari, Jamal Nasehifar, Alireza Malekzadeh, Afshin Ghaderpoury

**Affiliations:** a Safety Promotion and Injury Prevention Research Center (SPIPRC), Shahid Beheshti University of Medical Sciences, Department of Environmental Health Engineering, Tehran, Iran; b Nutritional Health Research Center, Lorestan University of Medical Sciences, Khorramabad, Iran; c Department of Environmental Health Engineering, School of Health and Nutrition, Lorestan University of Medical Sciences, Khorramabad, Iran; d Department of Environmental Health Engineering, School of Public Health, Zanjan University of Medical Sciences, Zanjan, Iran; e Students Research Committee, Shahid Beheshti University of Medical Sciences, Tehran, Iran

**Keywords:** Nitrate, Nitrite, Water quality, Dam

## Abstract

In recent years, contamination of water resources, with pollutants such as nitrate and nitrite, has significantly increased. These compounds can have harmful effects on human health, especially children such as methemoglobinemia. The main objective of this study was to measure the concentration of nitrate and nitrite and its health-risk assessment in the rivers entering Taham dam in Zanjan. USEPA Method was used to assess the health-risk of nitrate and nitrite. According to the obtained results, the concentration of nitrate and nitrite was in the range of 0.51–14.93 mg/l and 0.001–0.061 mg/l, respectively. According to the results, the mean of the CDI for nitrate and nitrite was 9.52*10^−2^ and 3.63*10^−4^ mg/kg/day, respectively. Furthermore, the mean HI for nitrate and nitrite was 5.97*10^−2^ and 3.63*10^−3^, respectively. The concentration of nitrate and nitrite in rivers was lower than the WHO and Iran guidelines. Based on the results, the HI value in all samples was less than 1 which indicating the non-carcinogenic effects of nitrate and nitrite in these rivers.

**Specifications Table**

**Table:** 

Subject area	*Chemistry, biology*
More specific subject area	*Water monitoring and quality*
Type of data	*Table, figure*
How data was acquired	*UV–vis spectrophotometer (DR-5000)*
Data format	*Raw, analyzed*
Experimental factors	*According to the study area, 36 sampling stations were identified. After sampling, all samples were stored in standard condition. Then, The concentration of nitrate and nitrite was measured*
Experimental features	*Measuring the concentration of*$NO_{3}^{-}$*and*$NO_{2}^{-}$*in the samples*
Data source location	*Zanjan city, Zanjan province, Iran*
Data accessibility	*Data are included in this article and supplemented excel file*

**Value of the data**

• Nitrate and nitrite are one of the most common pollutants of water resources. Therefore, its continuous monitoring is very important.
• One of its most important disadvantages is the formation of methemoglobinemia (blue baby), especially in children (< 6 months), which may have adverse effects.
• Dams are one of the main sources of water supply that are subject to various contaminants. Nitrogen compounds are one of these pollutants that can enter to water resources through agricultural sewage.
• One of the methods for assessing the effect of these compounds on human health is the health risk assessment.
• The data of this study shows the concentration of nitrate and nitrite in the Taham dam, so it can be considered in environmental planning.

## 1 Data

Zanjan is located in west of Tehran. According to the latest census, the city population is around 411,001 people. The study area is located northwest of Zanjan and has two main rivers (Golherod and Sarmesaghlo). These two rivers flow to Taham dam which is the main source of drinking water of Zanjan city.

## 2 Experimental design, materials, and methods

**Fig. 1:**
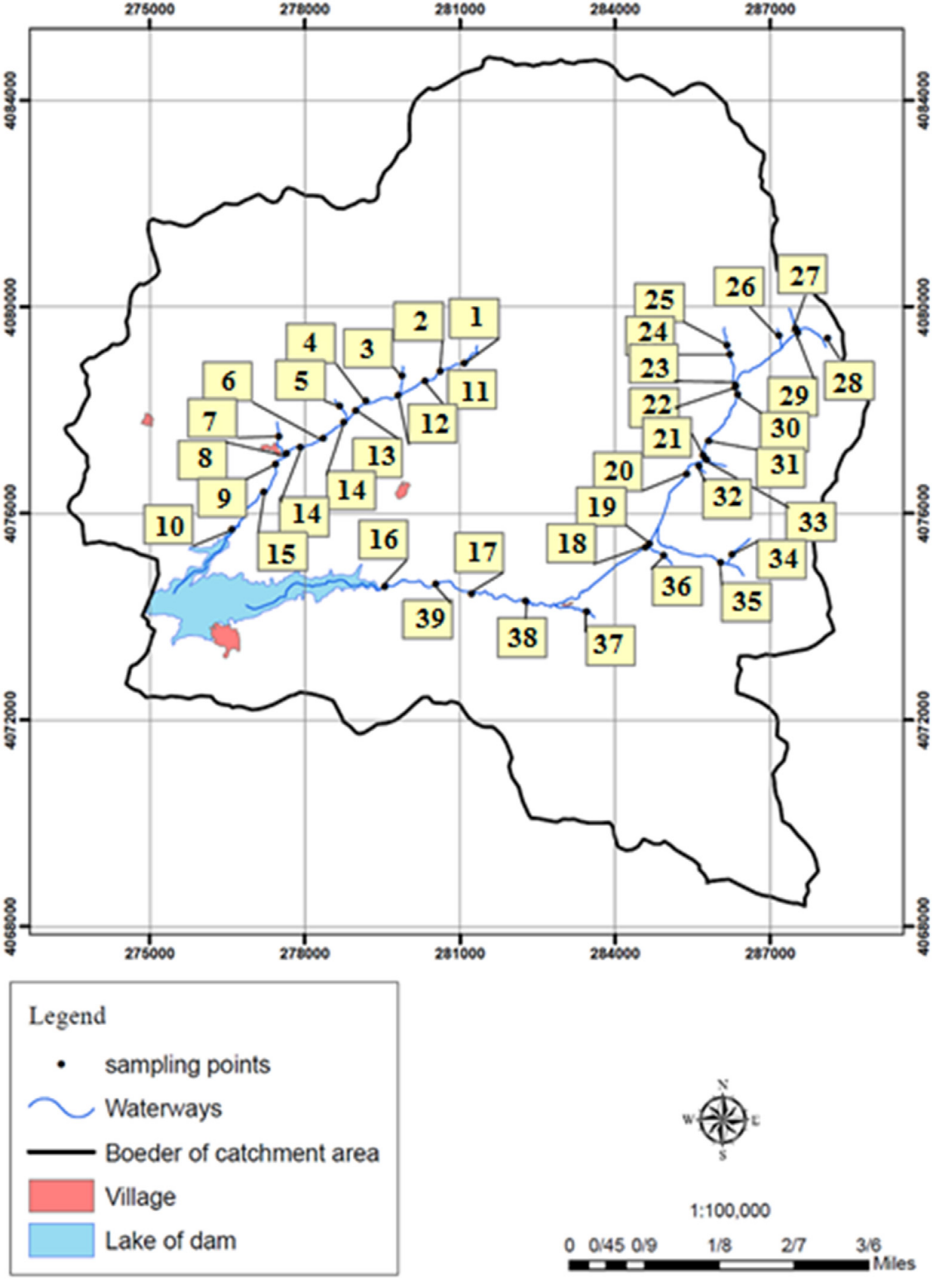
Sampling points in the studied area.

In recent years, contamination of water resources has increased significantly with pollutants, such as nitrate and nitrite. These compounds (nitrate and nitrite) can have harmful effects on human health [1]. In this study, 39 samples were taken through the study area. The sampling points illustrated in Fig. 1. After sampling, the samples were stored in standard condition for further analysis [2]. In this work, UV–vis spectrophotometer (DR-5000) was used to measure nitrate and nitrite concentration. USEPA Method was used to conduct the health-risk assessment associated with nitrate and nitrite. The following equation (Eq. (1)) was finally used to calculate the non-carcinogenic effects [3], [4], [5]:

(1) HI=CDI/RfD

where HI, CDI, and RfD are Hazard Index, Chronic Daily Intake (mg/kg/day), and Reference dose (mg/kg/day), respectively. Also, Eq. (2) was used to calculate the Chronic Daily Intake:

(2) $$CDI=(C_{W}{}^{*}DI)/\left(BW\right)$$

where *C*_W_ is nitrate and nitrite concentration in water (mg/l). DI is the average-daily intake of water (L/day). Also, BW is Body weight (kg). A HI value > 1 (more than 1) will show a significant risk level, where the higher the value, the greater the likelihood of adverse non-carcinogenic health effects. In this work, the applied RfD values for $NO_{3}^{-}$ and $NO_{2}^{-}$ were 1.6 and 0.1 mg/kg/day, respectively [6], [7], [8], [9], [10], [11], [12]. Table 1, Table 2 show the results of CDI and HI for nitrate and nitrite, respectively. The findings of the study showed that the mean concentration of nitrate and nitrite in the study area were 3.73 and 0.01 mg/l, respectively. Also, the maximum concentration of nitrate and nitrite was 14.93 and 0.06 mg/l. According to the results, the concentration of nitrate and nitrite in rivers flowing to the dam was lower than WHO guidelines and Iran standards. According to the results, the mean of the CDI for nitrate and nitrite were 9.52*10^−2^ and 3.63*10^−4^ mg/kg/day, respectively. Also, the maximum of the CDI for nitrate and nitrite was 3.83*10^−1^ and 1.56^*1^0^−3^ mg/kg/day, respectively. In addition it was revealed that the mean HI for nitrate and nitrite was 5.97*10^−2^ and 3.63*10^−3^, respectively. Based on the results, the HI value in all samples was less than 1 which indicating the non-carcinogenic effects of nitrate and nitrite in these rivers.

**Table 1:** The CDI and HI values for nitrate in Taham dam in Zanjan.

**Code**	**1**	**2**	**3**	**4**	**5**	**6**	**7**	**8**	**9**	**10**	**11**
$NO_{3}^{-}$	2.80E+00	2.75E+00	1.22E+01	5.13E−01	5.93E+00	5.45E+00	9.13E−01	2.63E+00	4.80E+00	2.13E+00	3.23E+00
**CDI**	7.18E−02	7.05E−02	3.13E−01	1.31E−02	1.52E−01	1.40E−01	2.34E−02	6.73E−02	1.23E−01	5.45E−02	8.27E−02
**HQ**	7.18E−02	7.05E−02	3.13E−01	1.31E−02	1.52E−01	1.40E−01	2.34E−02	6.73E−02	1.23E−01	5.45E−02	8.27E−02
**Code**	**12**	**13**	**14**	**15**	**16**	**17**	**18**	**19**	**20**	**21**	**22**
$NO_{3}^{-}$	2.53E+00	2.85E+00	3.13E+00	2.40E+00	4.75E+00	3.40E+00	4.15E+00	3.73E+00	1.49E+01	7.00E+00	3.90E+00
**CDI**	6.47E−02	7.31E−02	8.01E−02	6.15E−02	1.22E−01	8.72E−02	1.06E−01	9.55E−02	3.83E−01	1.79E−01	1.00E−01
**HQ**	6.47E−02	7.31E−02	8.01E−02	6.15E−02	1.22E−01	8.72E−02	1.06E−01	9.55E−02	3.83E−01	1.79E−01	1.00E−01
**Code**	**23**	**24**	**25**	**26**	**27**	**28**	**29**	**30**	**31**	**32**	**33**
$NO_{3}^{-}$	2.50E+00	3.13E+00	1.53E+00	1.88E+00	1.30E+00	1.75E+00	1.58E+00	1.65E+00	1.33E+00	1.30E+00	2.55E+00
**CDI**	6.41E−02	8.01E−02	3.91E−02	4.81E−02	3.33E−02	4.49E−02	4.04E−02	4.23E−02	3.40E−02	3.33E−02	6.54E−02
**HQ**	6.41E−02	8.01E−02	3.91E−02	4.81E−02	3.33E−02	4.49E−02	4.04E−02	4.23E−02	3.40E−02	3.33E−02	6.54E−02
**Code**	**34**	**35**	**36**	**37**	**38**	**39**	**40**	**41**	**42**		
$NO_{3}^{-}$	1.40E+00	1.48E+00	3.28E+00	6.58E+00	1.31E+01	5.55E+00	2.95E+00	2.40E+00	3.33E+00		
**CDI**	3.59E−02	3.78E−02	8.40E−02	1.69E−01	3.35E−01	1.42E−01	7.56E−02	6.15E−02	8.53E−02		
**HQ**	3.59E−02	3.78E−02	8.40E−02	1.69E−01	3.35E−01	1.42E−01	7.56E−02	6.15E−02	8.53E−02

**Table 2:** The CDI and HI values for nitrite in Taham dam in Zanjan.

**Code**	**1**	**2**	**3**	**4**	**5**	**6**	**7**	**8**	**9**	**10**	**11**
$NO_{2}^{-}$	1.75E−03	1.50E−03	5.00E−04	1.48E−02	5.50E−03	6.50E−03	6.00E−03	4.00E−03	7.50E−03	1.95E−03	7.50E−04
**CDI**	4.49E−05	3.85E−05	1.28E−05	3.78E−04	1.41E−04	1.67E−04	1.54E−04	1.03E−04	1.92E−04	5.00E−05	1.92E−05
**HQ**	4.49E−05	3.85E−05	1.28E−05	3.78E−04	1.41E−04	1.67E−04	1.54E−04	1.03E−04	1.92E−04	5.00E−05	1.92E−05
**Code**	**12**	**13**	**14**	**15**	**16**	**17**	**18**	**19**	**20**	**21**	**22**
$NO_{2}^{-}$	7.50E−04	2.00E−03	2.15E−02	1.55E−03	1.60E−02	6.08E−02	7.00E−03	6.00E−03	6.00E−03	5.55E−02	1.90E−02
**CDI**	1.92E−05	5.13E−05	5.51E−04	3.97E−05	4.10E−04	1.56E−03	1.79E−04	1.54E−04	1.54E−04	1.42E−03	4.87E−04
**HQ**	1.92E−05	5.13E−05	5.51E−04	3.97E−05	4.10E−04	1.56E−03	1.79E−04	1.54E−04	1.54E−04	1.42E−03	4.87E−04
**Code**	**23**	**24**	**25**	**26**	**27**	**28**	**29**	**30**	**31**	**32**	**33**
$NO_{2}^{-}$	1.90E−02	2.55E−02	3.20E−02	2.05E−02	1.55E−02	1.38E−03	2.05E−02	3.33E−02	2.80E−02	3.75E−03	2.93E−02
**CDI**	4.87E−04	6.54E−04	8.21E−04	5.26E−04	3.97E−04	3.53E−05	5.26E−04	8.53E−04	7.18E−04	9.62E−05	7.50E−04
**HQ**	4.87E−04	6.54E−04	8.21E−04	5.26E−04	3.97E−04	3.53E−05	5.26E−04	8.53E−04	7.18E−04	9.62E−05	7.50E−04
**Code**	**34**	**35**	**36**	**37**	**38**	**39**	**40**	**41**	**42**		
$NO_{2}^{-}$	2.75E−03	1.00E−03	5.43E−02	3.25E−02	1.65E−02	3.00E−03	4.50E−03	2.25E−03	3.25E−03		
**CDI**	7.05E−05	2.56E−05	1.39E−03	8.33E−04	4.23E−04	7.69E−05	1.15E−04	5.77E−05	8.33E−05		
**HQ**	7.05E−05	2.56E−05	1.39E−03	8.33E−04	4.23E−04	7.69E−05	1.15E−04	5.77E−05	8.33E−05		
