# Supplementary material for: Data on nitrate and nitrate of Taham dam in Zanjan (Iran)
Source: Data Brief. 2018 Feb 2;17:431–7. doi: 10.1016/j.dib.2018.01.055 (PMC5988385; doi:10.1016/j.dib.2018.01.055)
Supplement: Supplementary file 1 — Supplementary material [file mmc1.docx]

Dear Professor,

Editor of **Data in Brief**

The authors of this article declare that they have no conflict of interests.

**Data on health risk assessment of nitrate and nitrate into Taham dam in Zanjan, Iran**

Mohammadreza Masoudinejad, Mansour Ghaderpoori, Ali Jafari, Jamal Nasehifar, Alireza Malekzadeh, Afshin Ghaderpoury
